# Predicting Mechanical Strength of Alkali-Activated High-Performance Concrete Using Machine-Learning Methods

**DOI:** 10.3390/ma19112235

**Published:** 2026-05-25

**Authors:** Rahul Biswas, Farzin Kazemi, Akhilendra Sharma, Robert Jankowski, Panagiotis G. Asteris

**Affiliations:** 1 Department of Applied Mechanics, Visvesvaraya National Institute of Technology, Nagpur 440010, Maharashtra, India; rahulbiswas@apm.vnit.ac.in (R.B.); akhilendrasharma.er@gmail.com (A.S.); 2 Faculty of Civil and Environmental Engineering, Gdańsk University of Technology, ul. Narutowicza 11/12, 80-233 Gdansk, Poland; robert.jankowski@pg.edu.pl; 3 Department of Architecture, Built Environment and Construction Engineering, Politecnico di Milano, Piazza Leonardo da Vinci 32, 20133 Milan, Italy; 4 Computational Mechanics Laboratory, School of Pedagogical & Technological Education, GR-14121 Athens, Greece; panagiotisasteris@gmail.com

**Keywords:** alkali-activated high-performance concrete, environmental prospect, machine-learning algorithm, hyperparameter optimization, construction material

## Abstract

The growing demand for concrete poses a significant environmental challenge, but alkali-activated high-performance concrete (AA-HPC) offers a more sustainable alternative by potentially reducing carbon emissions and ecological harm. This study explores the latest developments in machine learning (ML) applications aimed at predicting the compressive strength of AA-HPC, with a focus on minimizing experimental expenses, construction duration, and environmental impact. Among nine evaluated ML models, the combination of extreme gradient boosting (XGBoost) with the African vultures optimization algorithm (AVOA) emerged as the most effective. AVOA proved highly efficient in optimizing model parameters, achieving the lowest root mean square error (RMSE) during hyperparameter tuning. On the training dataset, XGB-AVOA reached an R^2^ of 0.994 and an RMSE of 2.368, while on the testing dataset, it maintained superior performance with an R^2^ of 0.975 and an RMSE of 5.664. These findings highlight AVOA’s strength in fine-tuning XGBoost compared to alternative optimizers such as grey wolf optimizer (GWO), whale optimization algorithm (WOA), social spider optimization (SSO), and gorilla troops optimizer (GTO). To support practical implementation, a graphical user interface (GUI) has also been developed, allowing researchers to efficiently utilize the XGB-AVOA model for accurate, cost-effective, and time-saving predictions in laboratory environments.

## 1. Introduction

Alkali-activated high-performance concrete (AA-HPC) has emerged as a promising alternative to conventional cement-based materials in response to the urgent need for reducing the environmental impact of the construction sector, which is a major contributor to global greenhouse gas emissions. The production of ordinary Portland cement is highly energy-intensive and accounts for a significant share of anthropogenic CO_2_ emissions, thereby intensifying concerns related to climate change and resource depletion. In this context, AA-HPC utilizes alkali-activated binders derived from aluminosilicate sources such as fly ash (FA), ground granulated blast furnace slag (GGBFS), and metakaolin, which are industrial byproducts. These precursors react with alkaline activators, typically solutions of sodium or potassium hydroxide, silicate, or carbonate, through a process known as alkali activation, forming a hardened binder matrix. While AA-HPC offers significant potential for reducing reliance on clinker-based systems and improving resource efficiency, its environmental performance is not universally guaranteed and depends strongly on the specific formulation and type of activator used. Nevertheless, AA-HPC generally exhibits excellent mechanical properties, including high compressive strength and enhanced durability, along with strong resistance to chemical attack. These characteristics make it particularly suitable for demanding structural and aggressive environmental applications, such as infrastructure and marine engineering [1].

**Figure 1:**
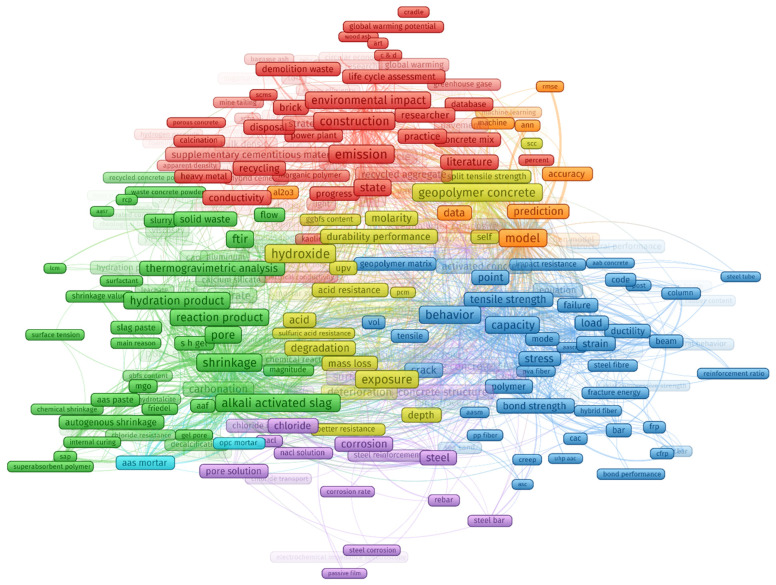
Collection of AA-HPC literature presented in main five categories.

The environmental benefits of alkali-activated cements and concretes are not universally guaranteed and depend strongly on the specific formulation and activator type. While these materials can reduce energy consumption and greenhouse gas emissions through the use of industrial byproducts and the avoidance of clinker production, recent life cycle assessment studies have shown that such advantages are conditional. In particular, systems employing high-impact activators (e.g., sodium silicate) may exhibit environmental burdens comparable to or even exceeding those of conventional Portland cement-based materials, whereas simplified formulations such as one-part geopolymers tend to demonstrate more consistent reductions in environmental impact [2]. Figure 1 presents a collection of 4342 works in the literature on AA-HPC that are presented in five main categories. As obvious, AA-HPC has a strong connection with recent trends in concrete materials.

AA-HPC is gaining recognition as a sustainable alternative compared with traditional Portland cement concrete. However, due to the wide variability in the composition of aluminosilicates and the inability of conventional materials science methods to establish reliable composition–property relationships, predicting the properties of AA-HPC has been challenging. Wetzel and Middendorf [3] explored the optimization of a Portland cement-free, alkali-activated material for enhanced strength and durability. Using GGBFS, potassium-based activators, silica fume, and metakaolin, the study achieved compressive strength comparable to that of UHPC while reducing capillary porosity. The low water-to-binder ratio and good workability were attained with specific silica fume amounts, even without the use of superplasticizers. Gomaa et al. [4] presented a random forest (RF) model for predicting key properties of FA-based AA-HPC, specifically slump flow and compressive strength. The model, after optimization, accurately predicts these properties based on physiochemical attributes, curing time, and mixing procedures.

Zhang et al. [5] developed a slag-based alkali-activated ultra high-performance concrete (UHPC) with ultra-low moisture content to address the CO_2_ emissions associated with traditional UHPC production. The optimized mix results in high compressive strength (i.e., 118 MPa at 28 days) and excellent hydration properties. The low moisture content enhances the fresh and mechanical properties of the UHPC, making it a promising material for achieving sustainable construction goals and meeting global carbon reduction targets. Cai et al. [6] investigated the durability characteristics of clinker-less UHPC made with alkali-activated binders, focusing on water absorption, carbonation, chloride diffusion, and corrosion resistance. The results show that while the material offers superior water absorption and chloride resistance compared to ordinary UHPC, it has weaker carbonation resistance. The corrosion rate of steel in alkali-activated binders is higher than in ordinary UHPC, possibly due to its higher moisture content. The study also suggests that steel fibers have limited influence on the durability of alkali-activated binders, indicating a well-bonded fiber–matrix interface. Zhang et al. [7] investigated alkali-activated UHPC made by replacing mineral powder with recycled concrete fines. The results show that alkali-activated UHPC, with a water-to-binder ratio of 0.29–0.27 and rice husk ash content of 10–30%, achieves a compressive strength of 120 MPa and has only 75% of the carbon emissions compared to conventional material.

## 2. Recent Trends in Machine Learning Models

**Figure 2:**
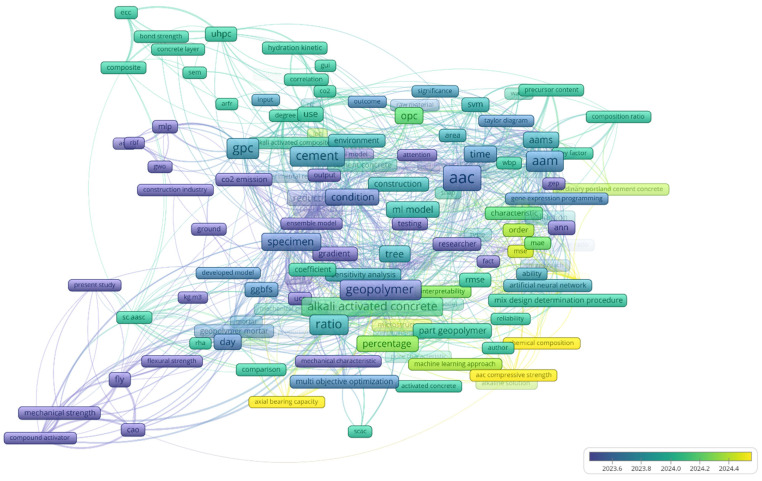
Collection of ML and AA-HPC literature presented in recent years.

Considering machine-learning (ML) methods and AA-HPC as the main keywords of research in the Web of Science database, only 72 papers were found to focus on this topic, as depicted in Figure 2. This shows the recent advances on this topic and the importance of using ML models for prediction of the mechanical properties of AA-HPC. Zhang et al. [8] introduced a chemistry-informed ML method for accurately estimating the compressive strength of alkali-activated materials using a dataset of 676 mixture designs. The model employs feature engineering to improve predictive performance and explores the impact of precursor and activator formulations on compressive strength. Shafighfard et al. [9] developed a stacked ML method for predicting compressive strength of AA-HPC using 538 experimental datasets. The ML method achieved 98% predictive performance, outperforming 18 other ML models; therefore, a graphical user interface (GUI) was also created to estimate AA-HPC compressive strength, cost, and carbon emissions, supporting further research and practical applications in the field.

The nature-inspired African vultures optimization algorithm (AVOA) emulates the scavenging behavior of African vultures. These vultures are known for their strategic teamwork, keen observation, and adaptability in searching for food. AVOA considers these characteristics to solve optimization problems by exploring and exploiting the search space efficiently. It is particularly useful in parameter tuning, feature selection, and solving high-dimensional problems due to its balance between exploration (i.e., diversifying the search space) and exploitation (i.e., intensifying the focus around promising solutions). Tested on standard benchmark functions and engineering problems, AVOA demonstrated outstanding performance, outperforming several existing algorithms [10]. Extreme gradient boosting (XGBoost), on the other hand, is a powerful and widely used ML algorithm based on gradient boosting frameworks. Known for its speed and performance, XGBoost handles large datasets efficiently and performs well on structured data. It is often employed in regression and ranking tasks due to its scalability and ability to reduce overfitting through regularization; moreover, a wide range of applications in engineering like prediction of vibration response, mechanical performance capacity, and failure probability of structures have also been developed. It is worth noting that the mechanical properties of concrete materials are widely explored in the literature due to having many input features that affect the results of the predictions [11].

Kaloop et al. [12] proposed a hybrid method for accurately estimating the shear strength of reinforced concrete (RC) deep beams. Support vector regression (SVR) was combined with three advanced metaheuristic optimization algorithms: AVOA, particle swarm optimization (PSO), and Harris Hawks optimization (HHO). The resulting hybrid models were developed and evaluated against existing models for performance comparison. The SVR-AVOA model outperformed others with a mean absolute error (MAE) of 43.17 kN and a maximum error of ±3.39%, making SVR-AVOA a powerful tool for accurate shear strength estimation. Ikram et al. [13] introduced an extreme learning machine network combined with the chaos red fox optimization algorithm to predict the shear strength of concrete beams reinforced with composite rebar. The model, validated against experimental data and design equations, demonstrated superior predictive performance and included sensitivity analysis to assess the impact of key parameters on beam performance. Wang et al. [14] introduced hybrid ML models combining support vector regression (SVR) with multi-objective artificial vultures optimization (MAVO), tunicate swarm algorithm (TSA) and AVOA to estimate the compressive strength of HPC. Among the models, SVM integrated with MAVO achieved the highest predictive performance (i.e., R^2^ = 0.968, RMSE = 2.19), highlighting the efficiency and cost-effectiveness of hybrid approaches for concrete design. Ding et al. [15] presented a hybrid approach combining SVR with AVOA to predict the compressive strength of HPC using 168 experimental samples. The SVR-AVOA model demonstrated superior predictive performance, highlighting its potential for enhancing HPC modeling. Zhang and Bai [16] investigated the radial basis function (RBF) model combined with AVOA and Salp swarm algorithm (SSA) to evaluate the compressive strength of HPC. The RBF-AVOA model achieved exceptional predictive performance, with R^2^ = 0.997 and MAE of 0.1917, showcasing its reliability and stability. Xiong et al. [17] proposed a novel approach by integrating Elman neural networks (ENNs) with the developed AVOA to predict the structure’s natural frequency. This combined method enables researchers and engineers to estimate the compressive strength of advanced concrete structures more accurately, paving the way for stronger and more reliable structural designs. Guo et al. [18] used AVOA-ENNs for predicting self-compacting concrete compressive strength. Using eight and 140 input parameters, the enhanced model achieved high predictive performance, with 140-parameter networks significantly improving prediction errors for 7-day and 28-day strengths. The study demonstrates AVOA’s efficiency and cost-effectiveness, highlighting its potential in civil engineering applications. Liang et al. [19] presented an interpretable boosting machine framework enhanced by AVOA for predicting diaphragm wall deflections in excavation projects. The hyperparameters of all models were optimized using AVOA, and the optimized models were then combined into a unified framework for a fair comparison. The AVOA-CatBoost model demonstrates exceptional performance, and an interpretable version has been developed to improve model transparency and practical utility. As presented in the literature, AVOA has superior ability to improve the hybrid ML model and improve its performance during predictions. Therefore, AVOA was selected in this study to improve ML models for the purpose of estimating the compressive strength of AA-HPC.

Although several studies have previously investigated ML models for predicting the compressive strength of concrete materials, this research focuses on the development and evaluation of metaheuristic optimization-based ML frameworks for AA-HPC. Unlike conventional and stacked ML approaches, which may perform well within a specific dataset but often exhibit limited adaptability across different data distributions, the proposed hybrid optimization strategy aims to enhance model robustness and generalization capability through systematic hyperparameter tuning and data-driven optimization. In particular, metaheuristic algorithms are employed to optimize the hyperparameters of base learners, enabling a more flexible and adaptive modeling framework capable of handling complex nonlinear relationships in heterogeneous experimental datasets. This approach also incorporates structured data preprocessing and validation strategies to improve model stability and reduce sensitivity to data variability. However, it is important to emphasize that the proposed models are intended as predictive and interpretive tools rather than replacements for experimental testing. While they can significantly reduce the reliance on extensive laboratory work by providing rapid and reliable estimations of compressive strength, their applicability remains bounded by the range and quality of the available data. Accordingly, the objective of this study is to propose and evaluate optimized ML models that improve predictive performance and generalization behavior for AA-HPC systems, thereby contributing to more efficient material design workflows and supporting future efforts toward reducing experimental cost and environmental impact in concrete development.

## 3. Data Preparation and Resampling

This section presents, in detail and depth, the database that was used for the training and reliability evaluation of the computational mathematical predictive models developed for predicting the mechanical strength of alkali-activated high-performance concretes. Particular emphasis is placed on the fundamental principles that should govern the composition of the database.

The compiled AA-HPC database was carefully examined to ensure transparency and reliability in both its material representation and statistical characteristics. The dataset, derived from multiple published experimental studies, was preprocessed through a structured screening procedure to ensure consistency in units, completeness of key variables, and physical plausibility of all records. Descriptive statistical analysis was performed to characterize the distribution of input parameters, while Pearson correlation coefficients were used as an initial exploratory tool to identify linear associations among variables. These correlations are interpreted cautiously as indicators of potential relationships rather than definitive causal links, particularly in the presence of possible multicollinearity inherent to heterogeneous experimental data. Based on the above principles for database compilation, an experimental database has been assembled using datasets on alkali-activated concrete, which were collected through an extensive literature review. The dataset consists of 18 input parameters, including steel fiber aspect ratio (SF_A_) and volume fraction (SF_V_), quartz powder (QP), SF, GGBS (Ground Granulated Blast-furnace Slag), water (W), solution-to-binder (S/B), flow (F), sodium metasilicate (SM), FA, sodium hydroxide (SH), curing temperature (T), sodium silicate (SS), quartz sand (QS), SS/SH, molarity (M), water-to-binder (W/B), and curing days (CD), whereas the output parameter of the dataset is the compressive strength (CS). The descriptive analysis of the dataset, as shown in Table 1, shows that the dataset consists of a wide range of variables that can be used for modeling. The authors used the dataset provided in [9]; however, the dataset was improved to 674 data points, including new sources published recently. The compiled database was first expanded to 674 data points and subsequently subjected to a structured data cleaning procedure to ensure consistency and reliability. Screening was conducted at the level of individual data points, with entire studies excluded only if all associated entries failed the defined criteria. Data points were removed if they (i) fell outside physically meaningful or experimentally plausible ranges for the variables considered, (ii) contained missing key variables that could not be reliably reconstructed, (iii) were identified as duplicates based on matching study descriptors and experimental conditions, or (iv) exhibited inconsistencies with the original sources, such as transcription or unit conversion errors. Following this process, 133 data points were excluded, resulting in a final curated dataset of 541 data points used for subsequent analyses. This method can improve the dataset and prevents outliers from being included and affecting the results.

The influence of key input parameters on the mechanical performance of AA-HPC should be interpreted in the context of material behavior rather than solely through statistical importance. Among these, curing time plays a fundamental role in strength development, as prolonged curing allows continued reaction progression and structural densification, leading to improved compressive and tensile strength. The binder composition, particularly GGBS and fly ash, significantly governs the stiffness and compactness of the hardened matrix; higher GGBS content is generally associated with enhanced early-age strength and reduced porosity. Water content directly affects the internal pore structure, where lower effective water-to-binder ratios promote a denser microstructure and higher compressive strength, while excessive water increases voids and weakens the matrix. The molarity of the alkaline activator influences the efficiency of the activation process, affecting the formation rate and distribution of binding phases, which in turn impacts both strength and durability. From a material design perspective, these parameters do not act independently but interact in a coupled manner, where optimal performance is achieved through a balanced combination rather than extreme values of individual variables. The trends observed in the present study are consistent with established findings in alkali-activated systems, where mechanical performance is strongly linked to matrix densification, reduced porosity, and improved interfacial bonding.

**Figure 3:**
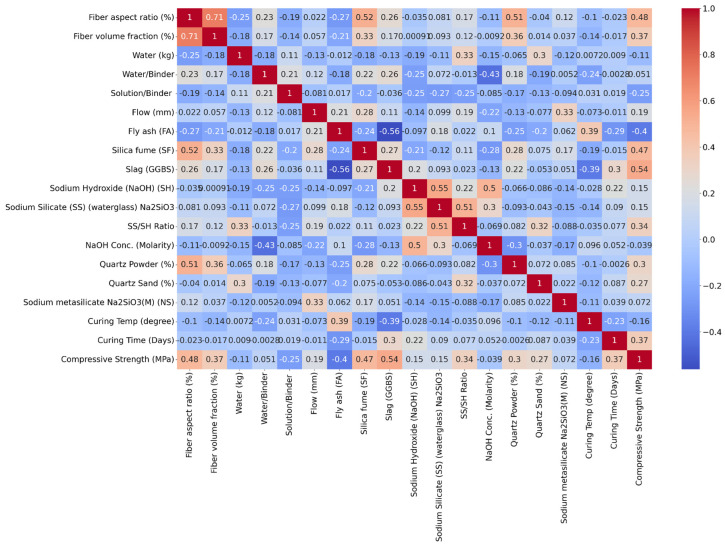
Pearson correlation matrix presented for modified dataset.

The Pearson correlation analysis was conducted to provide an initial exploratory assessment of the linear relationships among the input variables and the target output. In addition to correlation coefficients, statistical significance was evaluated using corresponding *p*-values based on the sample size. It was observed that most correlations are statistically significant at conventional levels (e.g., *p* < 0.05), which is expected given the relatively large dataset. However, due to the heterogeneous nature of the compiled data from multiple literature sources, the assumptions underlying classical significance testing such as independence and identical distribution of observations may not be fully satisfied. Therefore, the correlation results were interpreted cautiously and intended to provide qualitative insights into variable associations rather than definitive statistical inference. To better illustrate the correlations between the selected input features, a Pearson correlation matrix is shown in Figure 3. The Pearson correlation indicates the positive and negative effects of input features on compressive strength. It is seen that the slag has the highest positive impact (0.54) on the compressive strength, followed by SF_A_ (0.48), SF (0.47), and T (0.37), respectively, whereas FA has the highest negative (−0.4) impact, followed by the S/B (−0.25) and T (−0.016), respectively. The SM has the lowest effect on the compressive strength parameter, with a value of 0.072. The overall study suggests that the input parameters have a considerable effect on compressive strength, and hence, they should be selected as inputs for prediction models.

The computational framework for model development and analysis was implemented in the Python programming environment (Python 3.11). The ML models and data processing workflows were developed using widely adopted scientific libraries, including scikit-learn, XGBoost 3.2.0, NumPy 2.4.6, pandas 3.0.3, Matplotlib 3.8.4, and Seaborn 0.13.2. All simulations and optimization procedures were executed on a personal computer equipped with an Intel Core i7 processor (2.3–3.5 GHz) and 32 GB of RAM. This consistent computational setup enabled stable and comparable evaluation of all models.

## 4. Conventional ML Models

Conventional ML algorithms have been developed so far to be used for engineering problems. However, they were improved by new techniques and modern computers to be used easily. The most important decision tree algorithm is random forest (RF), for which multiple datasets are prepared by resampling the original dataset with replacement, allowing each dataset to be slightly different. Then, each of these datasets trains a separate decision tree and random feature selection at each node of the trees, resulting in uncorrelated decision trees. Aggregation combines predictions from all the trees by averaging in regression, which helps in cancelling out individual model errors and improves RF’s ability for estimating compressive strength [20,21].

The concept of boosting was first discussed by Schapire, which further led to the development of the first boosting algorithm, AdaBoost, in 1995. In the adaptation of AdaBoost for regression, the model is designed to improve predictive performance by combining multiple weak learners through sequential training and adjusting weights. AdaBoost is very adaptable and easy to implement because of its iterative approach; however, it is susceptible to outliers and noisy data, which could result in overfitting.

To overcome this issue, gradient boosting machine (GBM) was developed, which works by using the gradient descent algorithm to minimize errors and continuously updating the initial prediction with improved estimates. The final model is a combination of all the preceding predictions, each adjusted with appropriate weights. A GBM model can be represented as

(1) $$f_{M}\left(x_{j}\right)=\sum_{m}^{M}\gamma_{m}h_{m}\left(x_{j}\right)$$

where *M* is number of trees, *h_m_* is a weak learner, and *γ_m_* is a scaling factor.

GBM is suitable to handle complex patterns but is prone to overfitting in noisy datasets. This model is best suited when working with smaller datasets [22,23]. Therefore, XGBoost was developed, which has ascended to become the most dominant ML tool in the field of research and data science competition [24,25]. XGBoost works on the principle of the GBM model, of iteratively combining the set of weak learners to minimize the error by optimizing the loss function *L_M_* at each step. The objective function to be optimized includes two parts:

(2) $$\mathrm{Loss}\;\mathrm{function}:\;L\left(y_{i},F\left(x_{i}\right)\right)={\left(y_{i}-F\left(x_{i}\right)\right)}^{2}$$

(3) $$\mathrm{Regularization}:\;\Omega \left(f\right)=\gamma T+\frac{1}{2}\lambda \sum_{j=1}^{T}w_{j}^{2}$$

The objective function is

(4) $$L_{M}\left(F\left(x_{i}\right)\right)=\sum_{i=1}^{n}L\left(y_{i},F\left(x_{i}\right)\right)+\sum_{m=1}^{M}\Omega \left(f_{k}\right)$$

where *y_i_* is the actual value for the *i*th sample in the dataset, *F*(*x_i_*) is the predicted value for the *i*th sample at iteration t, and *f_k_* is the *k*th tree. Further, as XGBoost uses second-order Taylor approximation of the objective function in Equation (4), this can considerably improve the ability of XGBoost for estimating the compressive strength of concrete, as discussed in detail [26]. The overfitting problem of the GBM model is addressed in XGBoost primarily because of the introduction of a regularization term, and it is also capable of handling missing values. XGBoost can be further enhanced in its predictive performance by carefully tuning its hyperparameters, as shown in some studies that focused on hyperparameter optimization to improve their predictive models. To ensure robust model development and mitigate overfitting, the hyperparameter optimization process was conducted within a k-fold cross-validation (CV) framework (i.e., *k* = 5), whereby model performance was evaluated across multiple folds of the training data. In addition, the dataset was partitioned into distinct training, validation, and testing subsets, with the testing set kept entirely unseen during both the training and optimization processes. To further prevent information leakage, data points originating from the same literature source were assigned to the same subset, avoiding overlap of closely related experimental observations across different phases. This combined strategy of CV, strict data separation, and source-wise grouping provides a more reliable assessment of model generalization and ensures that the reported performance metrics reflect true predictive capability rather than overfitting to the training data.

## 5. Metaheuristic Optimization Methods

In this research, five metaheuristic optimization methods were implemented to improve the performance of the XGBoost method. The successful implementation of metaheuristic optimization methods for hyperparameter optimization, mechanical properties of self-compacting concrete, axial load capacity of concrete-filled steel tube column [27], and the carbonation depth of FA concrete were investigated by the authors. Moreover, in this research, improving the XGBoost method with novel metaheuristic optimization methods could endow it with more robust strength for unseen datasets and enhance its ability. Therefore, this research focused on approaches to be used for the implementation of such methods and developing them.

### 5.1. Grey Wolf Optimizer

**Figure 4:**
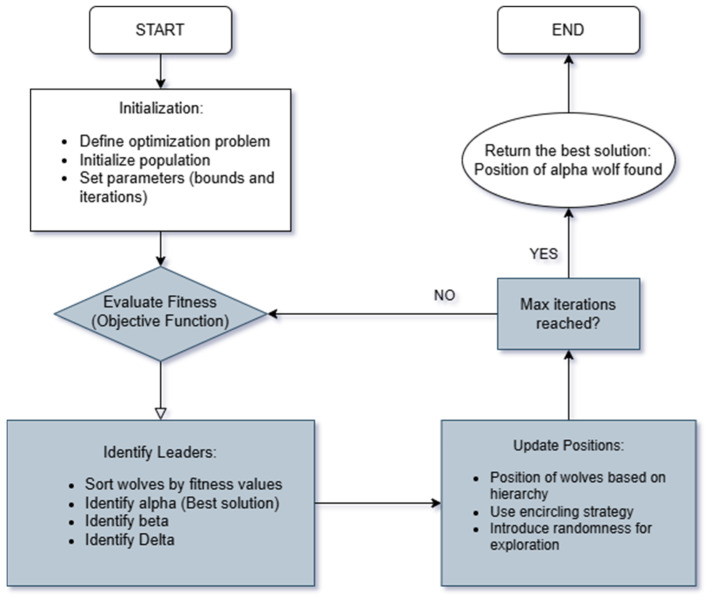
Flowchart presentation of GWO for optimization process.

The grey wolf optimizer (GWO) was developed to mimic the rank-based hunting mechanism of the apex predators, grey wolves [28,29]. The leaders in their hierarchy are called alpha wolves, which manage the pack and are responsible for decision-making. Alpha wolves lead the hunt, supported by the assistance of beta wolves, with delta and omega wolves following behind. The hunt starts with the exploration phase, where the wolves diverge from each other, locate the prey, and start encircling it; alpha wolves have the best knowledge of the potential location of the prey followed by beta and delta wolves. Based on these positions for the optimum location of prey, other wolves update their position. This is known as the exploitation phase, where the wolves converge for the attack. Once the prey stops moving, the attack begins. This process is mathematically simulated and used for the optimization process, whose workflow is given in Figure 4.

### 5.2. Whale Optimization Algorithm

**Figure 5:**
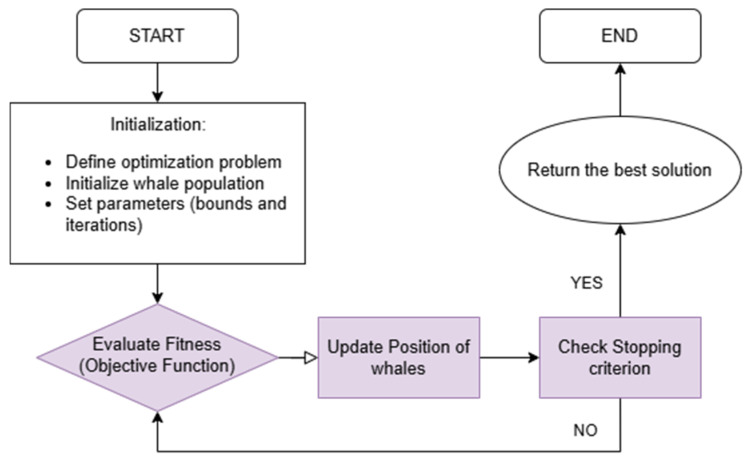
Flowchart presentation of WOA for optimization process.

The whale optimization algorithm (WOA), depicted in Figure 5, was developed by Mirjalili et al. [30] to simulate the bubble-net hunting strategy of humpback whales. Whales locate (i.e., exploration phase) and surround their prey (i.e., typically krill or small fish herds) and create upward spirals of bubbles to attack their prey. This process involves two main strategies: shrinking and encircling, where whales converge towards the prey, and spiral movement, where whales move in a helical pattern. The whales explore the search space by moving randomly when they are farther from the prey. It is shown that the hybrid models can increase the performance of conventional ML models [31].

### 5.3. Social Spider Optimization Algorithm

**Figure 6:**
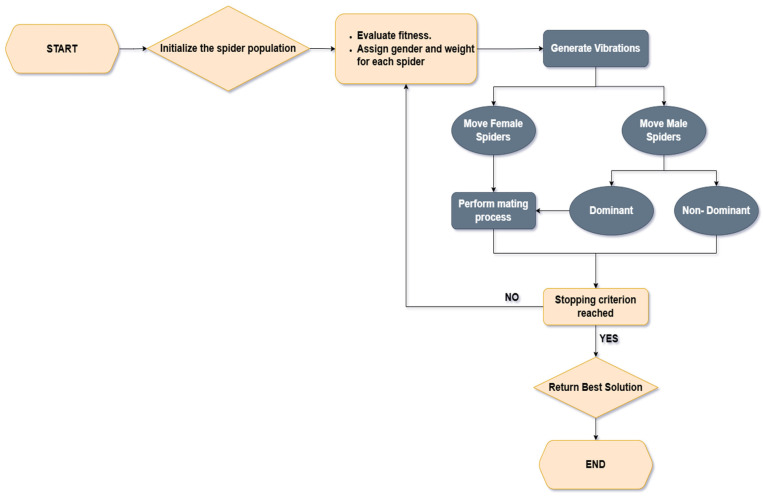
Flowchart presentation of SSO for optimization process.

The social spider optimization (SSO) algorithm is a nature-inspired metaheuristic optimization technique modeled after the foraging behavior of spiders in a social web. In SSO, the population represents spiders, which are divided into two groups (i.e., male and female) to simulate natural biological roles. Each spider communicates with others through vibrations on the web, which represent the fitness of their positions in the search space. Spiders move toward promising regions based on these vibrations while balancing exploration and exploitation to optimize a given objective function [32]. The flowchart of the SSO algorithm is depicted in Figure 6.

Integrating SSO with XGBoost to create a hybrid model can enhance predictive performance by considering the optimization capabilities of SSO for hyperparameter tuning in XGBoost. This hybrid implementation involves using SSO to search for the optimal combination of XGBoost hyperparameters (e.g., learning rate, maximum depth, and number of estimators) that maximize model predictive performance or minimize error on a validation dataset. The process begins with initializing a population of spiders, each representing a potential hyperparameter configuration. The fitness of each spider is evaluated by training and validating an XGBoost model with its corresponding hyperparameters. Based on fitness, spiders adjust their positions iteratively, refining the hyperparameters until convergence or a predefined number of iterations. This hybrid approach combines the robust optimization capability of SSO with the high-performance learning framework of XGBoost, yielding a model with improved predictive performance and efficiency.

### 5.4. African Vultures Optimization Algorithm

**Figure 7:**
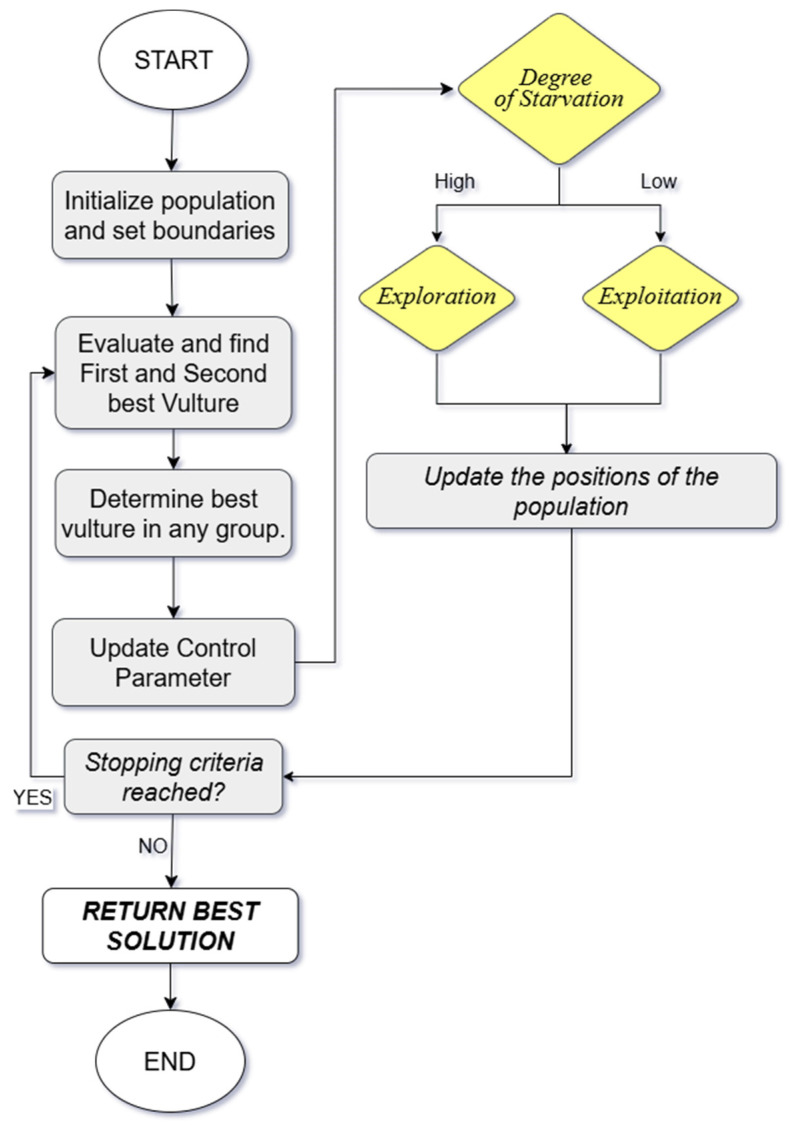
Flowchart presentation of AVOA for optimization process.

AVOA is inspired by the competitive scavenging behavior of African vultures and known for its simplicity, adaptability and exceptional performance. The flowchart of the AVOA algorithm is shown in Figure 7. AVOA models the vultures’ strategy of travelling long distances upon discovering food conflicts among different species, which are eventually dominated by the stronger vultures.

The weaker vultures encircle the stronger ones, waiting for their turn to feed. The process begins with initializing a population of vultures, followed by evaluation of the fitness function of each vulture based on an objective function. After evaluating fitness, the algorithm identifies the two best vultures. Exploration continues until the vultures are exhausted, and the vultures that have begun starving stay close to the vultures that have found food. Vultures explore new areas to locate food (i.e., solutions) by using two strategies: moving based on the leaders’ positions while considering starvation and random exploration within specified boundaries to ensure diversity and avoid premature convergence. To use AVOA, the process uses random factors to balance exploitation (intensive search near good solutions) and exploration (broad search for new solutions). Exploitation takes place in two stages: namely, the siege-fight stage and siege-strife stage. In the siege-fight stage, the stronger vultures dominate the weaker ones without sharing food, while the weaker vultures compete using rotating flight behavior, thereby provoking and exhausting the stronger ones. In the siege-strife stage, vultures converge on a single food source and engage in violent siege-strife. The two best vultures compete with each other for the remaining food [15,16]. These behaviors allow AVOA to effectively search a solution space, making it suitable for complex optimization problems. To implement AVOA with XGBoost to evaluate the compressive strength of AA-HPC with 17 input features, the algorithm can be used to optimize XGBoost’s hyperparameters. A population of vultures was selected as a potential solution, where each vulture’s position corresponds to a set of hyperparameters for the XGBoost model (e.g., learning rate, max depth, gamma, number of estimators). The dataset was partitioned into three mutually exclusive subsets, namely training (70%), validation (15%), and testing (15%) sets. The training set was used to fit the XGBoost model, while the validation set was employed within the AVOA optimization loop to assess candidate hyperparameter configurations based on the MSE. The test set was strictly held out during model development and used only once for the final evaluation of the optimized model. To enhance robustness and reproducibility, data splitting was conducted using a fixed random seed, and the optimization process was repeated multiple times to ensure the stability of the obtained results. Furthermore, to prevent potential information leakage arising from the compiled nature of the dataset, all data points originating from the same literature source were assigned to the same subset, thereby avoiding overlap of related experimental observations across training, validation, and testing sets. This grouping strategy ensures a more reliable assessment of the model’s generalization capability. By combining the capabilities of AVOA with XGBoost’s powerful predictive performance, the hybrid approach aims to achieve high performance in predicting the compressive strength of AA-HPC.

### 5.5. Gorilla Troops Optimizer

The gorilla troops optimizer (GTO) is a metaheuristic algorithm inspired by the social and adaptive behaviors of gorillas in the wild. It mimics the dynamics of gorilla troops, such as their leadership hierarchy, group movement patterns, and strategies for resource exploitation. The algorithm models gorillas’ cooperative and competitive interactions to effectively balance exploration (i.e., searching new areas in the solution space) and exploitation (i.e., refining solutions in promising regions). GTO is well-suited for solving complex optimization problems due to its ability to adapt dynamically to varying solution landscapes [33].

To create a hybrid model, GTO can be utilized for hyperparameter tuning in XGBoost. A population of gorillas is generated, with each gorilla representing a candidate set of hyperparameters for XGBoost (e.g., learning rate, max depth, subsample). Then, the XGBoost model is trained using the hyperparameters of each gorilla and its performance on the validation set is evaluated based on metrics like MSE. GTO explores the solution space through random movements (i.e., representing the gorillas’ search behavior) and converge towards the best solutions found (i.e., mimicking resource exploitation). The positions of gorillas (i.e., hyperparameters) are updated iteratively based on the best-performing individuals and social dynamics of the troop. The algorithm stops when a stopping criterion (e.g., a predefined number of iterations or minimal fitness improvement) is met. Finally, the optimal hyperparameters discovered by GTO are used to train the final XGBoost model. Therefore, in this research, GTO helped optimize the hyperparameters of the XGBoost model trained on 17 input features, and provided new insights on hyperparameter tuning.

## 6. Results and Discussion

### 6.1. Hyperparameter Optimization

The main step of analysis is hyperparameter tuning based on the optimization methods described in Section 4. As illustrated, the methods can be implemented on XGBoost and help to find the best hyperparameters of the model; this can provide an automated approach to be used for any other dataset provided. However, in this research, the AA-HPC dataset was used to present the proposed ML models. Tuning the five hyperparameters enlisted in Table 2 was performed by an optimized XGBoost model to minimize Root Mean Squared Error (RMSE).

**Figure 8:**
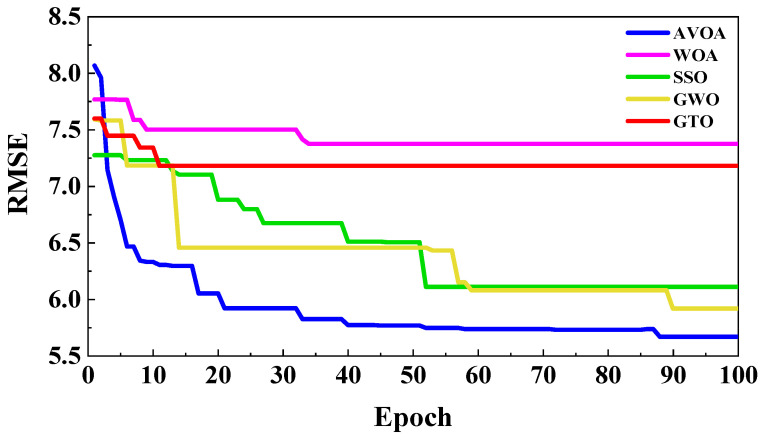
Convergence graph for optimizers used to find the best hyperparameters.

Owing to the complexity of tuning the hyperparameters through trial-and-error methods to achieve the best results, metaheuristic algorithms were used to evaluate the best combination of hyperparameters. Metaheuristics are nature-inspired advanced optimization techniques that balance exploration and exploitation to find near-optimal solutions, albeit a global optimum. Metaheuristic algorithms are categorized primarily into six categories: physics-based, mathematics-based, evolution-based, bio-based, human-based, and swarm-based. However, in this research, the advanced swarm-based algorithms of GWO, WOA, SSO, AVOA, and GTO were developed to be implemented in XGBoost [34,35]. These optimizers were run over 100 iterations with a population size of 50. After the completion of all iterations, the best fitness (minimum RMSE) and best solutions (hyperparameters) corresponding to those RMSE values were recorded as the output. The convergence of the RMSE over the number of iterations is depicted in Figure 8. As presented, the AVOA is the best optimization algorithm to reduce the RMSE of the model in the designated number of iterations. The SSO and GWO algorithms attempted to reduce the RMSE, with GWO obtaining a lower value compared to SSO and showing better performance for the optimization. WOA and GTO were not able to reduce the RMSE, and they had higher values compared to other optimization methods. Table 3 lists the range of hyperparameters set as bounds, along with their optimal values corresponding to the best fitness of each optimizer. They were used in improved ML methods for evaluating the compressive strength of AA-HPC.

### 6.2. Shapley Additive Explanations

**Figure 9:**
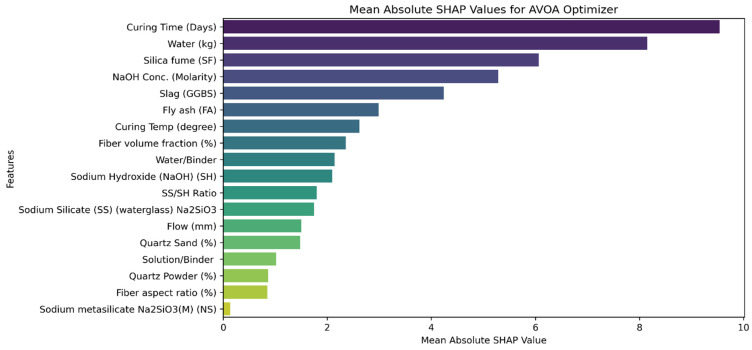
Mean SHAP values for feature importance effects.

**Figure 10:**
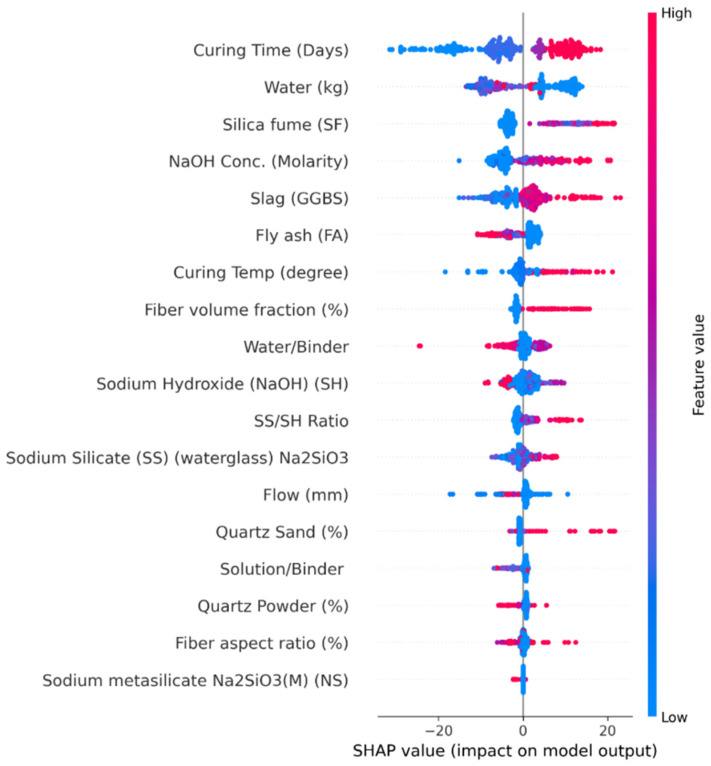
SHAP impact values on XGB-AVOA model.

Shapley additive explanations (SHAP) is a feature selection method used to evaluate the contribution of each feature to the model’s prediction. It has been demonstrated that SHAP values are superior to alternative approaches in terms of providing computing efficiency, matching human intuition, and guaranteeing consistent performance across many model types. In regression problems, SHAP works by breaking down a model’s prediction into the individual contributions of each feature, such as how much each feature influences the final value. It does this by calculating the average impact of each feature across all possible combinations of other features, ensuring that each feature’s contribution is fairly accounted for. SHAP focuses on those important features that affect the compressive strength of AA-HPC. Curing time plays a decisive role in strength evolution, as extended curing allows for continued reaction progress and structural refinement of the binder matrix. This results in improved load transfer within the material, leading to higher compressive strength. Water content significantly influences strength by controlling the internal pore structure; lower effective water content generally leads to a denser matrix, which improves compressive strength. However, excessive reduction in water may hinder proper reaction progression, negatively affecting strength development; therefore, the range of water content, as shown in the SHAP details in Figure 9 and Figure 10, plays a critical role. The molarity of the activating solution directly affects the efficiency of the activation process, which in turn governs the formation and distribution of binding phases. An optimal molarity contributes to a more homogeneous and compact matrix, thereby improving compressive strength through better internal bonding. Slag content has a pronounced effect on mechanical performance, as higher slag proportions typically result in a denser and more refined microstructure. These interpretations provide a clearer physical basis for the SHAP results, linking the relative importance of input variables to their direct influence on the mechanical strength characteristics of AA-HPC. SHAP values can help to understand the importance of both the global and local explanations. In this study, the best performing model, AVOA-XGBoost, was selected for SHAP analysis. As depicted in Figure 9, the input features were ranked according to their average contribution across all predictions by the mean absolute SHAP values, highlighting that curing time is the most influential feature, followed by water, SF, and molarity.

In contrast, input features like SM and SF_A_ showed minimal impact. The directionality of these effects is shown in Figure 10, a SHAP summary plot in the form of bee swarm plots, where feature values are represented by color coding (i.e., blue for low and red for high) and the horizontal spread of SHAP values shows the range of impact. It can be observed that the model’s output primarily increases with increasing curing time and decreases with lower values. The balanced contribution of GGBS, with both high and low values having a positive and negative impact on forecasts, indicates that it plays a diverse role in output variability. Conversely, W/B exhibits a clear trend whereby greater values (red) generally result in consistently higher model output. Similarly, depending on their particular levels, characteristics like FA and M exhibit mixed effects.

### 6.3. Predicting Compressive Strength of AA-HPC

**Figure 11:**
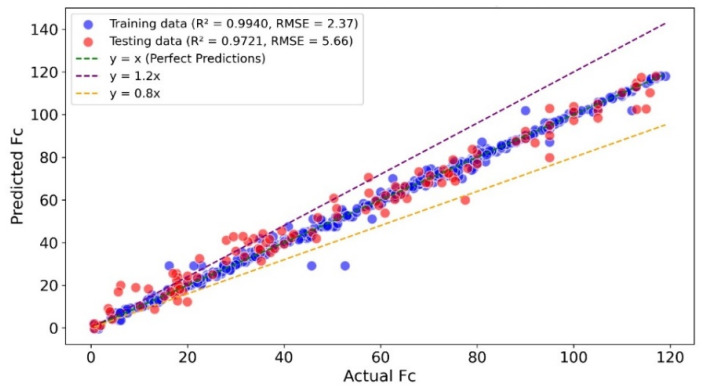
Actual vs. predicted results for compressive strength of AA-HPC using XGB-AVOA.

**Figure 12:**
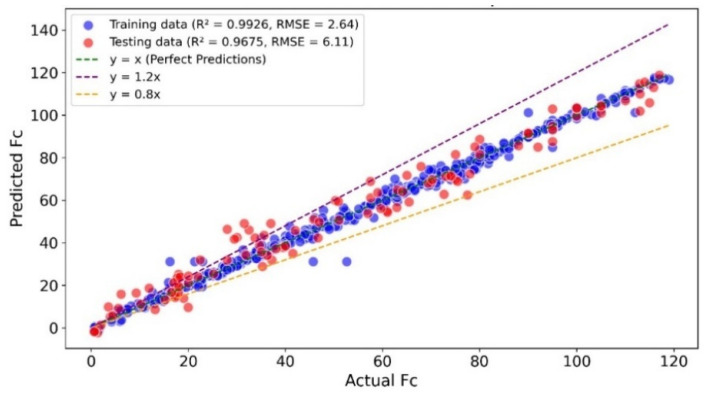
Actual vs. predicted results for compressive strength of AA-HPC using XGB-SSO.

**Figure 13:**
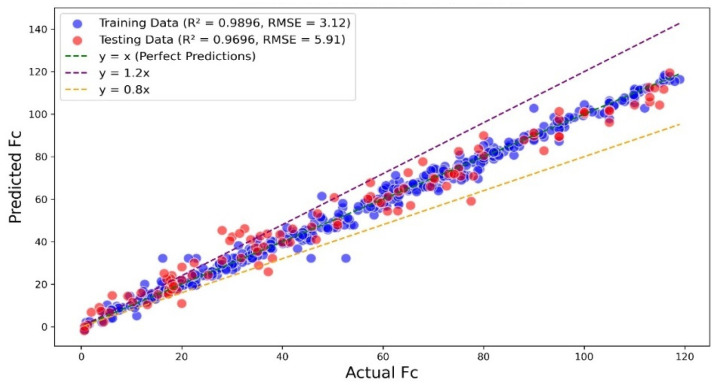
Actual vs. predicted results for compressive strength of AA-HPC using XGB-GWO.

The scatter plots for the actual versus training phases of the three best-performing models are presented in Figure 11, Figure 12 and Figure 13. As presented, the proposed ML models aimed to estimate the compressive strength of AA-HPC in different ranges up to 120 MPa. It can be observed that the proposed ML models had better fitting values for compressive strength of more than 80 MPa; however, for lower values, there was some divergence around the x = y line. Meanwhile, the predictive performance of more than 98% assured their ability in estimating the compressive strength.

Table 4 lists metrics of the performance evaluation of the ML models during both training and testing phases. The performance metrics reveal notable differences in predictive performance and generalization capability among the models. In the training phase, XGB-AVOA outperformed all other models, achieving the highest R^2^ value of 0.994. Similarly, it yielded the lowest RMSE (i.e., 2.368) and Ratio of the Root Mean Square Error to the Standard Deviation of Measured Data (RSR) (i.e., 0.077), indicating minimal error and high consistency. The robustness of the model was further supported by its low Weighted Mean Absolute Percentage Error (WMAPE) (i.e., 0.022) and high Nash-Sutcliffe efficiency coefficient (NS) (i.e., 0.994) values. In contrast, the AdaBoost model showed the weakest performance, with the lowest R^2^ of 0.913 and highest RMSE of 9.308, suggesting limited capability in capturing the complexity of the dataset. Other optimized XGBoost variants, such as XGB-GTO and XGB-WOA, also demonstrated strong training performance with only minor variations across evaluation metrics, highlighting the adaptability of XGBoost when coupled with different optimization strategies. However, a clearer distinction among models emerged in the testing phase. While the conventional XGBoost model achieved excellent training predictive performance (with R^2^ = 0.995), its performance declined noticeably on the test set (with R^2^ = 0.834, RMSE = 14.224), indicating a tendency toward overfitting. In contrast, the optimized XGBoost models maintained both high predictive performance and strong generalization. Among them, XGB-AVOA was identified as the best-performing model, achieving the highest R^2^ of 0.975 and the lowest RMSE of 5.664 along with superior values for Variance Accounted For (VAF) (i.e., 97.302), RSR (i.e., 0.167), and Willmott’s Index (WI) (i.e., 0.993). These results demonstrate its ability to generalize effectively to unseen data, making it a reliable predictive tool. Other optimized variants, including XGB-GWO and XGB-SSO, also exhibited strong performance, with R^2^ values of 0.971 and 0.969, respectively, and relatively low RMSE values, confirming the effectiveness of metaheuristic-based hyperparameter tuning. The RF model, although performing well during training, showed a substantial reduction in predictive performance during testing (with R^2^ = 0.859, RMSE = 13.011), further indicating overfitting or limited adaptability to unseen data. Similarly, GBM achieved moderate performance (with R^2^ = 0.877) but was still outperformed by the optimized XGBoost models.

The noticeable gap between training and testing performance observed for several baseline models is primarily attributed to differences in generalization capability and the absence of explicit hyperparameter tuning for optimal regularization. In models such as RF and standard XGBoost, high training accuracy reflected strong fitting capacity; however, this was accompanied by reduced test performance, indicating that the models captured dataset-specific patterns and noise rather than fully generalizable relationships. This behavior is characteristic of tree-based ensemble methods when model complexity (e.g., tree depth, number of estimators, and learning rate) is not optimally constrained. Since all models were evaluated under identical data partitioning and experimental conditions, the observed improvements can be attributed to the effectiveness of the optimization strategies rather than differences in data handling. The consistent and superior performance of XGB-AVOA across both training and testing phases highlights its robustness and suitability for modeling complex relationships in AA-HPC compressive strength prediction. These findings underscore the importance of advanced hybrid ensemble approaches in achieving both high predictive performance and reliable generalization.

### 6.4. Error Histogram

**Figure 14:**
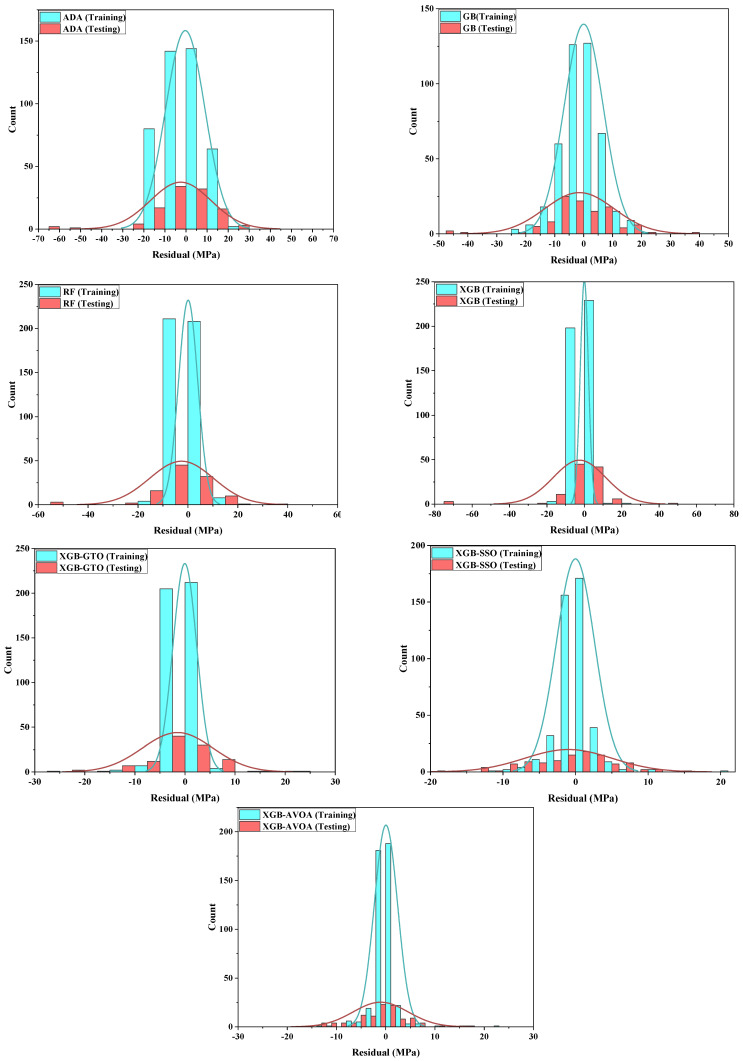
Error histogram of hybridized ML models.

An error histogram is a tool used to visualize the distribution of errors in an ML model’s predictions, particularly in regression models. It plots the error values on the x-axis, which are the differences between predicted and actual values, while the y-axis shows the frequency of these errors. The bins around zero error represent predictions that are close to accurate, while errors far from zero indicate larger inaccuracies. A well-performing model will generally have most errors concentrated near zero, suggesting good predictive performance. If the histogram is symmetrical, it shows balanced predictions, while a skewed histogram means the model either over-predicts or under-predicts consistently. A narrow spread of errors reflects reliable predictions, while a wider spread indicates variability and inconsistent performance. In Figure 14, the models’ performance was evaluated based on residual errors, and XGB-AVOA was found to be the most successful model, with the error ranging from −15% to +20%. The lowest residual error showed that this model was better at fitting the data. Based on their residual errors following XGB-AVOA, the models were arranged as XGB-SSO, XGB-GTO, XGB-WOA, and XGB-GWO.

Among the traditional ML techniques, XGBoost performed better than RF, GBM, and AdaBoost in terms of residual error, indicating its adaptability to the given data. The error of XGBoost for training data, with a narrow spread of errors, reflected reliable predictions, whereas the error for testing data ranged from −60% to +40%, indicating a wider spread and variability and inconsistent performance. The observation also validates the result of previous discussions. Overall, these results show hybrid optimization techniques, such as AVOA, can enhance XGBoost models’ predictive power while also providing useful data regarding the relative performance of other ML algorithms.

### 6.5. Taylor Diagram Presentation for ML Models

**Figure 15:**
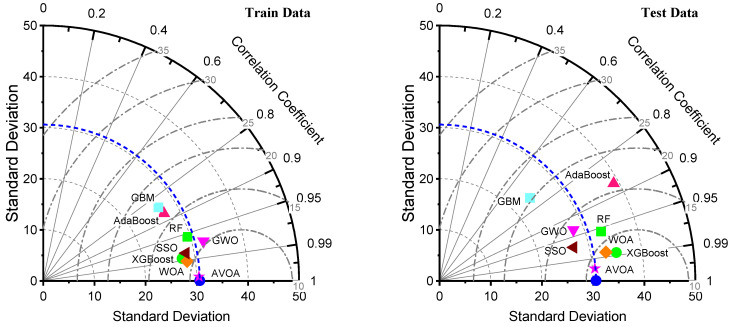
Taylor diagram for training and testing datasets using ML models.

The Taylor diagram is a powerful statistical tool developed to visualize relationships among several models. By combining three statistical metrics such that the distance from the reference point indicates the RMSE, the angle between the axes provides the correlation coefficient, and the radial distance from the origin displays the standard deviation, it is simple to determine which model most closely reflects the reference dataset by showing several models on a single Taylor diagram. In the training phase, as depicted in Figure 15, it can be observed that the XGB-AVOA, XGB-WOA, and XGBoost models, which had the highest predictive performance, are the ones that are closest to the reference point. However, GBM and AdaBoost were out of bounds and had less predictive performance than the other models. Although the training part is important in learning how well the ML models train, the Taylor diagram in the testing part is very informative regarding the capability of the proposed ML models in estimating the compressive strength of AA-HPC. As presented in Figure 15, only XGB-AVOA was near the reference point, and the other ML models were far from it. However, the XGB-WOA and XGBoost models had better performance compared to the other ML models, and GBM and AdaBoost had the worst performance. The results confirm that XGBoost variations are still competitive, and conventional ML models such as GBM, RF, and AdaBoost perform noticeably worse.

### 6.6. Graphical User Interface for Practical Engineering

**Figure 16:**
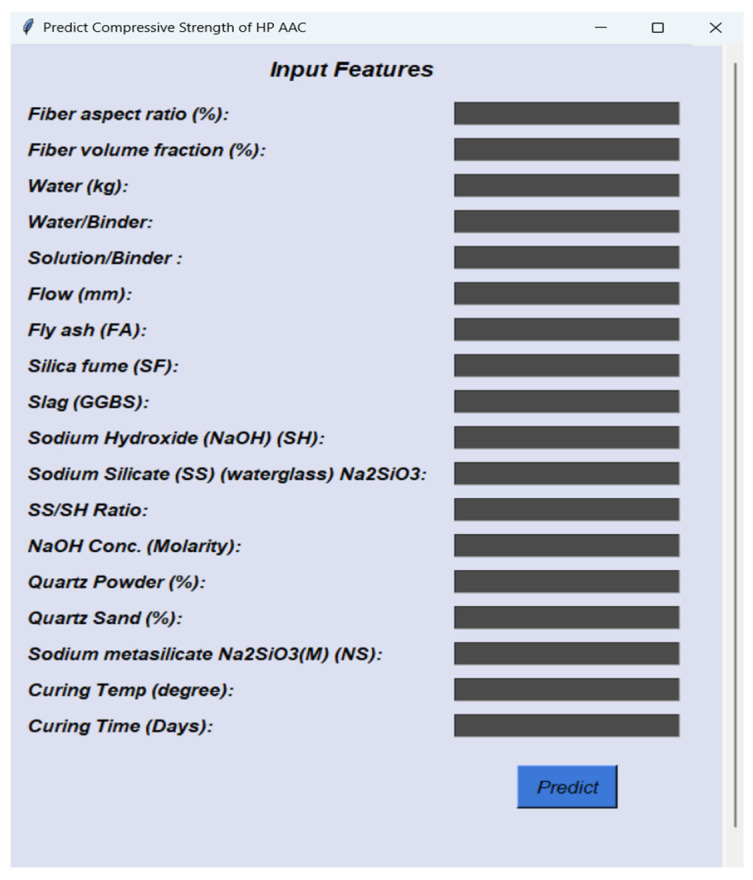
GUI for predicting compressive strength of AA-HPC.

Structural engineers find ML models less beneficial due to a growing dependency on long-term forecasts based on lab experiments, which often disregard empirical approaches. Designers need essential access to those techniques for validation, advancement, and continuous improvement. This study addresses this gap by introducing an effective ML model capable of estimating the compressive strength of AA-HPC. To provide accurate and comprehensible predictions with lower computational demands, the present work developed a public GUI. The user-friendly interface uses the XGB-AVOA model implemented in Tkinter to evaluate the compressive strength of AA-HPC, as shown in Figure 16. In the preliminary prediction phase, users engage with this interface to enter essential parameters that correspond to the ranges found in the datasets used for training the ML models. The screen interface displays the calculated compressive strength values with the user-defined input parameters. The function automatically executes upon any modification of inputs. The prediction process is remarkable for its rapidity, usually terminating in a few seconds. This work, using SHAP interpretation and an intuitive GUI prediction tool, confirmed the significant influence of curing time on the compressive strength of AA-HPC. Results confirmed a need for prolonged curing periods to achieve optimum strength growth in AA-HPC. The GUI application enables users to quickly understand the importance of extended curing, hence improving practical knowledge in concrete engineering.

## 7. Limitations and Future Research

Every optimal computational predictive model that has been developed and proposed must be used with caution, as its domain of applicability is strictly defined by the bounds of the input parameters employed during its training, specifically between their minimum and maximum observed values. Similarly, in the present study, the proposed GUI, based on the optimal model for predicting the mechanical strength of AA-HPC, is valid only within the parameter ranges reported in Table 1, and within the compressive strength interval of approximately 1 to 120 MPa represented in the dataset. Although the database comprises a substantial number of records and the resulting model demonstrates high predictive reliability, it should be emphasized that its use is intended for interpolation within the validated feature space, and extrapolation beyond these limits may lead to unreliable predictions due to the absence of supporting experimental data. In this context, the GUI is designed as a practical implementation tool to facilitate the application of the trained model within its defined domain, rather than as a universal predictive system. One of the authors’ immediate research directions is the further expansion of the database by incorporating additional experimental data, particularly in underrepresented regions of the input space. This will enable the development of a more comprehensive and balanced dataset, thereby improving the statistical coverage of the feature domain. Ultimately, retraining the model on an expanded dataset is expected to enhance its predictive performance, robustness, and generalization capability, contributing to a deeper understanding of the mechanical behavior of AA-HPC.

## 8. Conclusions

This research focused on optimization-based ML models to considerably improve their performance by automating hyperparameter tuning for the improved adoptability of ML models. Then, a dataset for AA-HPC was prepared to check the capability of the proposed ML models improved by five optimization algorithms: GWO, WOA, SSO, AVOA, and GTO. The main results of the research are detailed below:

- The best performing model, AVOA-XGBoost, was selected for SHAP analysis, and the input features were ranked according to their average contribution across all predictions by the mean absolute SHAP values. Curing time was the most influential feature, followed by water, SF, and molarity. In contrast, input features like SM and SF_A_ showed minimal impact. However, according to the heatmap presentation, the input features had a Pearson correlation relationship and should be considered in the analysis.
- Owing to the complexity of tuning the hyperparameters through a trial-and-error method to achieve best results, metaheuristic algorithms were used to evaluate the best combination of hyperparameters. Considering 100 iterations with a population size of 50, the best fitness (i.e., minimum RMSE) and best solutions (i.e., hyperparameters) were determined by XGB-AVOA. AVOA was the best optimization algorithm for reducing the RMSE of the model in the designated iteration number. GWO yielded a lower value compared to SSO and better performance for optimization; however, WOA and GTO were not able to reduce the RMSE, and they had higher values compared to the other optimization methods.
- In the training set, XGB-AVOA surpassed all other models, achieving the highest R^2^ value of 0.994 and the lowest RMSE (2.368) and RSR (0.077) values. The robustness of the model on the training data was further supported by the WMAPE and NS values, which were 0.022 and 0.994, respectively. XGB-GTO and XGB-WOA showed strong performance with little variation in metrics like R^2^, RMSE, and VAF, which highlights XGBoost’s adaptability when combined with other optimization techniques.
- In the testing set, XGB-AVOA was identified as the superior model, exhibiting the highest R^2^ value (0.975), the lowest RMSE (5.664), and the lowest WMAPE (0.081). XGB-GWO and XGB-SSO demonstrated high R^2^ values of 0.971 and 0.969, respectively. GBM had an R^2^ value of 0.877, slightly better than the RF model with an R^2^ of 0.859. Therefore, in managing an unseen dataset, XGB-AVOA outperformed the other ML models on a dataset characterized by complex relationships.
- According to the Taylor diagram, it can be seen that XGB-AVOA, XGB-WOA, and XGBoost models, which had the highest predictive performance, were close to the reference point in the training set. On the testing set, only XGB-AVOA was near the reference point and was the best model, whereas the XGB-WOA and XGBoost models had better performance compared to other ML models.
- To utilize the achievements of this study and the proposed ML models, a GUI was proposed for use in predicting the compressive strength of AA-HPC. The user-friendly interface uses the XGB-AVOA model implemented in Tkinter and executes automatically upon any changes to the input variables. This tool can be used by experimental laboratories to reduce experiment costs and constructional time.

## Figures and Tables

**Table 1:** Descriptive metrics of the AA-HPC dataset using 541 data points.

**Metric**	**SF_A_ (%)**	**SF_V_ (%)**	**W (kg)**	**W/B**	**S/B**	**F (mm)**	**FA (kg)**	**SF (kg)**	**GGBS (kg)**	**SH (kg)**
Mean	35.78	0.50	42.84	0.34	0.73	86.71	43.47	12.18	50.90	14.29
Std.	34.27	0.66	54.31	0.11	0.50	103.95	37.08	8.64	23.90	11.33
Min	0	0	6.09	0.16	0.35	3.81	0	0	0	0.35
0.25	0	0	20.90	0.3	0.42	9.53	13.17	5.12	35.57	5.50
0.50	37.5	0	23.47	0.356	0.46	15.24	44.22	11.95	55.56	9.15
0.75	66.69	1	34.39	0.38	1.576	155.13	68.89	19.73	70	26.99
Max	125	2	275	1	1.576	360	278.80	52.67	99.19	37.5
Metric	SS (kg)	SS/SH	M	QP (kg)	QS (kg)	SM (kg)	T (°C)	CD (days)	Compressive Strength (MPa)	
Mean	17.23	1.87	7.27	14.12	32.87	3.16	29.68	17.72	55.41	
Std.	18.41	1.59	4.15	6.20	15.53	2.31	14.01	20.20	31.35	
Min	0	0.2	0	4.65	5	0.53	17.5	1	0.6	
0.25	9.54	1	3	10	14.46	2.4	23	7	28.9	
0.50	11.68	1.5	7.5	12.29	35.45	2.4	25	7	57.49	
0.75	17.01	2.5	10	18	43.20	2.4	25	28	79	
Max	141.76	7.72	16	26.94	56.92	8	90	90	119	

**Table 2:** Hyperparameters selected from XGBoost model for optimization.

Hyperparameter	Range	Default	Description
learning_rate	[0, 1]	0.3	Controls contribution of each tree to final model.
n_estimators	[0, ∞)	100	Increasing the number of trees allows modeling for enhanced predictions by adding corrections with each tree.
max_depth	[0, ∞)	6	Controls the maximum depth of the decision trees. A deeper tree can model more complex patterns but also risks overfitting, especially on small datasets.
subsample	(0, 1]	1	The proportion of data that will be randomly sampled for each tree.
Colsample_bytree	(0, 1]	1	Controls the fraction of features (columns) that are randomly sampled for each tree.

**Table 3:** Optimized hyperparameters according to optimization methods.

Hyperparameter	Range	Best Solution (i.e., Hyperparameters)				
		GWO	WOA	SSO	AVOA	GTO
learning_rate	[0.01, 0.3]	0.283367	0.218507	0.262831	0.29819	0.27334
n_estimators	[50, 1000]	457	79	838	998	856
max_depth	[3, 10]	3	7	3	3	3
subsample	[0.5, 1]	0.61005	0.69408	0.60576	0.71495	0.61324
Colsample_bytree	[0.5, 1]	0.65975	0.59632	0.61282	0.80991	0.60145

**Table 4:** Performance evaluation of ML models for training and testing phases.

**Training**	**RF**	**AdaBoost**	**GBM**	**XGBoost**	**XGB-GWO**	**XGB-WOA**	**XGB-GTO**	**XGB-SSO**	**XGB-AVOA**
R^2^	0.985	0.913	0.952	0.995	0.990	0.993	0.994	0.993	0.994
WMAPE	0.048	0.141	0.093	0.015	0.037	0.025	0.022	0.028	0.022
NS	0.984	0.907	0.951	0.995	0.990	0.993	0.994	0.993	0.994
RMSE	3.840	9.308	6.763	2.168	3.118	2.511	2.369	2.637	2.368
VAF	98.42	90.74	95.10	99.50	98.96	99.32	99.39	99.25	99.40
RSR	0.126	0.305	0.221	0.071	0.102	0.082	0.078	0.086	0.077
WI	0.991	0.941	0.971	0.997	0.997	0.998	0.997	0.996	0.997
Testing	RF	AdaBoost	GBM	XGBoost	XGB-GWO	XGB-WOA	XGB-GTO	XGB-SSO	XGB-AVOA
R^2^	0.859	0.827	0.877	0.834	0.971	0.957	0.958	0.969	0.975
WMAPE	0.163	0.203	0.167	0.140	0.087	0.102	0.096	0.092	0.081
NS	0.853	0.814	0.873	0.824	0.970	0.953	0.955	0.968	0.972
RMSE	13.011	14.604	12.091	14.224	5.914	7.373	7.178	6.107	5.664
VAF	85.826	81.988	87.466	82.982	97.009	95.364	95.703	96.840	97.302
RSR	0.384	0.431	0.357	0.420	0.174	0.217	0.212	0.180	0.167
WI	0.959	0.943	0.964	0.954	0.992	0.987	0.988	0.992	0.993

## Data Availability

The original contributions presented in this study are included in the article. Further inquiries can be directed to the corresponding author.

## References

[1] Pierrehumbert R. (2019). There is no Plan B for dealing with the climate crisis. Bull. At. Sci..

[2] Ouellet-Plamondon C., Habert G. (2015). Life cycle assessment (LCA) of alkali-activated cements and concretes. Handbook of Alkali-Activated Cements, Mortars and Concretes.

[3] Wetzel A., Middendorf B. (2019). Influence of silica fume on properties of fresh and hardened ultra-high performance concrete based on alkali-activated slag. Cem. Concr. Compos..

[4] Gomaa E., Han T., ElGawady M., Huang J., Kumar A. (2021). Machine learning to predict properties of fresh and hardened alkali-activated concrete. Cem. Concr. Compos..

[5] Zhang X.Y., Yu R., Zhang J.J., Shui Z.H. (2022). A low-carbon alkali activated slag based ultra-high performance concrete (UHPC): Reaction kinetics and microstructure development. J. Clean. Prod..

[6] Cai R., Tian Z., Ye H. (2022). Durability characteristics and quantification of ultra-high strength alkali-activated concrete. Cem. Concr. Compos..

[7] Zhang X.Y., Fan M.X., Zhou Y.X., Ji D.D., Li J.H., Yu R. (2023). Development of a sustainable alkali activated ultra-high performance concrete (A-UHPC) incorporating recycled concrete fines. J. Build. Eng..

[8] Zhang L.V., Marani A., Nehdi M.L. (2022). Chemistry-informed machine learning prediction of compressive strength for alkali-activated materials. Constr. Build. Mater..

[9] Shafighfard T., Kazemi F., Asgarkhani N., Yoo D.Y. (2024). Machine-learning methods for estimating compressive strength of high-performance alkali-activated concrete. Eng. Appl. Artif. Intell..

[10] Abdollahzadeh B., Gharehchopogh F.S., Mirjalili S. (2021). African vultures optimization algorithm: A new nature-inspired metaheuristic algorithm for global optimization problems. Comput. Ind. Eng..

[11] Asgarkhani N., Kazemi F., Lasowicz N., Jankowski R. (2026). Evaluation of mechanical behavior of concrete-timber-filled steel tubes using multi-stage machine-learning model. Structures.

[12] Kaloop M.R., Roy B., Chaurasia K., Kim S.M., Jang H.M., Hu J.W., Abdelwahed B.S. (2022). Shear strength estimation of reinforced concrete deep beams using a novel hybrid metaheuristic optimized SVR models. Sustainability.

[13] Ikram R.M.A., Dai H.L., Al-Bahrani M., Mamlooki M. (2022). Prediction of the FRP reinforced concrete beam shear capacity by using ELM-CRFOA. Measurement.

[14] Wang L. (2024). Estimating high-performance concrete compressive strength with support vector regression in hybrid method. Multiscale Multidiscip. Model. Exp. Des..

[15] Ding B., Wang Q., Ma Y., Shi H. (2024). Prediction of compressive strength of concrete for high-performance concrete using two combined models, SVR-AVOA and SVR-SSA. Multiscale Multidiscip. Model. Exp. Des..

[16] Zhang Y., Bai Z. (2024). Using radial basis function for estimating the high-performance concrete compressive strength with optimizing AVOA and SSA. J. Intell. Fuzzy Syst..

[17] Xiong T., Fan G., Khairy Y. (2024). Predicting the compressive strength of advanced concrete structures by developed African vulture optimization algorithm-Elman neural networks. Mechanics of Advanced Materials and Structures.

[18] Guo S., Kou H., Bi Y., Mamlooki M. (2024). Predicting the compressive strength of self-compacting concrete by developed African vulture optimization algorithm-Elman neural networks. Sci. Rep..

[19] Liang Z., Lin S., Dong M., Cao X., Guo H., Zheng H. (2024). Interpretable gradient boosting based ensemble learning and African vultures optimization algorithm optimization for estimating deflection induced by excavation. Frontiers of Structural and Civil Engineering.

[20] Han Q., Gui C., Xu J., Lacidogna G. (2019). A generalized method to predict the compressive strength of high-performance concrete by improved random forest algorithm. Constr. Build. Mater..

[21] Li Q.F., Song Z.M. (2022). High-performance concrete strength prediction based on ensemble learning. Constr. Build. Mater..

[22] Xu Y., Ahmad W., Ahmad A., Ostrowski K.A., Dudek M., Aslam F., Joyklad P. (2021). Computation of high-performance concrete compressive strength using standalone and ensembled machine learning techniques. Materials.

[23] Lee S., Nguyen N.H., Karamanli A., Lee J., Vo T.P. (2023). Super learner machine-learning algorithms for compressive strength prediction of high-performance concrete. Struct. Concr..

[24] Alabdullah A.A., Iqbal M., Zahid M., Khan K., Amin M.N., Jalal F.E. (2022). Prediction of rapid chloride penetration resistance of metakaolin based high strength concrete using light GBM and XGBoost models by incorporating SHAP analysis. Constr. Build. Mater..

[25] Nguyen H., Vu T., Vo T.P., Thai H.T. (2021). Efficient machine learning models for prediction of concrete strengths. Constr. Build. Mater..

[26] Feng D.C., Liu Z.T., Wang X.D., Chen Y., Chang J.Q., Wei D.F., Jiang Z.M. (2020). Machine learning-based compressive strength prediction for concrete: An adaptive boosting approach. Constr. Build. Mater..

[27] Kazemi F., Asgarkhani N., Ghanbari-Ghazijahani T., Jankowski R. (2025). Ensemble machine learning models for estimating mechanical curves of concrete-timber-filled steel tubes. Eng. Appl. Artif. Intell..

[28] Mirjalili S., Mirjalili S.M., Lewis A. (2014). Grey wolf optimizer. Adv. Eng. Softw..

[29] Ahmed H.U., Mostafa R.R., Mohammed A., Sihag P., Qadir A. (2023). Support vector regression (SVR) and grey wolf optimization (GWO) to predict the compressive strength of GGBFS-based geopolymer concrete. Neural Comput. Appl..

[30] Mirjalili S., Lewis A. (2016). The whale optimization algorithm. Adv. Eng. Softw..

[31] Qiu Y., Zhou J., Khandelwal M., Yang H., Yang P., Li C. (2022). Performance evaluation of hybrid WOA-XGBoost, GWO-XGBoost and BO-XGBoost models to predict blast-induced ground vibration. Eng. Comput..

[32] Cuevas E., Cienfuegos M., Zaldívar D., Pérez-Cisneros M. (2013). A swarm optimization algorithm inspired in the behavior of the social-spider. Expert Syst. Appl..

[33] Hussien A.G., Bouaouda A., Alzaqebah A., Kumar S., Hu G., Jia H. (2024). An in-depth survey of the artificial gorilla troops optimizer: Outcomes, variations, and applications. Artif. Intell. Rev..

[34] Asgarkhani N., Kazemi F., Jankowski R., Formisano A. (2025). Dynamic ensemble-learning model for seismic risk assessment of masonry infilled steel structures incorporating soil-foundation-structure interaction. Reliab. Eng. Syst. Saf..

[35] Asgarkhani N., Kazemi F., Jankowski R. (2025). Machine-learning based tool for seismic response assessment of steel structures including masonry infill walls and soil-foundation-structure interaction. Comput. Struct..

